# The diagnostic value of serum 25-hydroxyvitamin D, MIS, and insulin-like growth factor 1 for polycystic ovary syndrome (PCOS)

**DOI:** 10.5937/jomb0-62488

**Published:** 2026-06-16

**Authors:** Shaomeng Sun, Jianrui Zheng, Ziying Xie

**Affiliations:** 1 Tongji University School of Medicine, Department of Obstetrics and Gynaecology, Shanghai, China; 2 The First Affiliated Hospital of Zhengzhou University, Obstetrics and Gynaecology, Zhengzhou City, China

**Keywords:** polycystic ovary syndrome, 25-hydroxyvitamin D, insulin-like growth factor 1, insulin-like growth factor binding protein 3, anti-Mullerian hormone, sindrom policističnih jajnika, 25-hidroksivitamin D, insulinu sličan faktor rasta 1, protein koji vezuje insulinu sličan faktor rasta 3, anti-Milerov hormon

## Abstract

**Background:**

To investigate blood levels and the importance of Müllerian Inhibiting Substance (MIS), insulin-like growth factor 1 (IGF-1), insulin-like growth factor binding protein 3 (IGFBP-3), and 25-hydroxyvitamin D S25(OH)DC in patients with polycystic ovarian syndrome (PCOS).

**Methods:**

The observation group consisted of 212 PCOS patients who received treatment at this institution between May 2022 and May 2024, while the control group consisted of 212 healthy women who were physically examined at the same hospital during the same time frame. The two groups' general information, serum 25(OH)D, MIS, IGF-1, and IGFBP-3 levels, and sex hormones and glucose metabolism markers were compared. PCOS patients were divided into the IR group and the non-IR group based on the presence of insulin resistance (IR). The levels of serum 25(OH)D, MIS, IGF-1, and IGFBP-3 were compared between the two groups. The study examined the associations among the four markers and the homeostasis model of insulin resistance index (HOMA-IR), as well as their supplementary diagnostic utility for PCOS.

**Results:**

The level of 25(OH)D in the observation group was lower than that in the control group (P&lt; 0.05). Total testosterone (TT), luteinising hormone (LH), estradiol (E2), fasting insulin (FINS), fasting blood glucose (FBG), serum MIS, IGF-1, IGFBP-3, HOMA-IR, and the average body mass index were all greater in the observation group than in the control group (P&lt; 0.05). The IR group's 25(OH)D level was lower than the non-IR group's (P&lt; 0.05). Serum MIS, IGF-1, and IGFBP-3 levels were higher in the IR group than in the non-IR group (P&lt; 0.05). The levels of serum MIS, IGF-1 and IGFBP-3 in the observation group were positively correlated with HOMA-IR (r= 0.659, 0.586, 0.565, P&lt; 0.05), and 25(OH)D was negatively correlated with HOMA-IR (r=-0.548, P&lt; 0.05). The combined auxiliary diagnosis of PCOS by serum 25(OH)D, MIS, IGF-1, and IGFBP-3 had an area under the curve of 0.963, according to the results of receiver operating characteristic curve analysis, which was greater than that of the single detection of each index (0.819, 0.864, 0.814, 0.799).

**Conclusions:**

MIS, IGF-1, IGFBP-3, and serum 25(OH)D levels are associated with the development and occurrence of PCOS. The combined detection of these four indicators has high auxiliary diagnostic value for PCOS and is conducive to clinical prevention and treatment.

## Abstract (sr)

**Uvod:**

Cilj je bio da se ispitaju nivoi u krvi i značaj Milerove inhibirajuce supstance (MIS), insulinu sličnog faktora rasta 1 (IGF-1), proteina koji vezuje insulinu sličan faktor rasta 3 (IGFBP-3) i 25-hidroksivitamina D [25(OH)D] kod pacijentkinja sa sindromom policističnih jajnika (PCOS).

**Metode:**

Posmatrana grupa je obuhvatala 212 pacijentkinja sa PCOS koje su lečene u ovoj ustanovi u periodu od maja 2022. do maja 2024. godine, dok je kontrolna grupa obuhvatala 212 zdravih žena koje su fizički pregledane u istoj bolnici u istom periodu. Upoređeni su opšti podaci, serum 25(OH)D, MIS, IGF-1 i IGFBP-3, kao i polni hormoni i markeri metabolizma glukoze između dve grupe. Pacijentkinje sa PCOS su podeljene u IR grupu i ne-IR grupu na osnovu prisustva insulinske rezistencije (IR). Upoređeni su nivoi seruma 25(OH)D, MIS, IGF-1 i IGFBP-3 između ove dve grupe. Takođe, istražene su korelacije između četiri markera i indeksa homeostaze insulinske rezistencije (HOMA-IR), kao i njihova dopunska dijagnostička vrednost za PCOS.

**Rezultati:**

Nivo 25(OH)D u posmatranoj grupi bio je niži nego u kontrolnoj grupi (P&lt; 0,05). Ukupni testosteron (TT), luteinizirajuci hormon (LH), estradiol (E2), insulin natašte (FINS), glukoza u krvi natašte (FBG), serum MIS, IGF-1, IGFBP-3, HOMA-IR i prosečan indeks telesne mase bili su veći u posmatranoj grupi u odnosu na kontrolnu grupu (P&lt; 0,05). Nivo 25(OH)D u IR grupi bio je niži nego u ne-IR grupi (P&lt; 0,05). Nivoi seruma MIS, IGF-1 i IGFBP-3 u posmatranoj grupi bili su pozitivno povezani sa HOMA-IR (r= 0,659, 0,586, 0,565, P&lt; 0,05), dok je 25(OH)D bio negativno povezan sa HOMA-IR (r=-0,548, P&lt; 0,05). Kombinovana dopunska dijagnoza PCOS pomoću seruma 25(OH)D, MIS, IGF-1 i IGFBP-3 imala je površinu ispod krive (AUC) od 0,963, što je više od pojedinačne detekcije svakog indeksa (0,819, 0,864, 0,814, 0,799).

**Zaključak:**

Nivoi MIS, IGF-1, IGFBP-3 i serumski 25(OH)D su povezani sa razvojem i pojavom PCOS. Kombinovana detekcija ova četiri indikatora ima visoku dopunsku dijagnostičku vrednost za PCOS i doprinosi kliničkoj prevenciji i lečenju.

## Introduction

Polycystic ovary syndrome (PCOS) has an incidence rate of 5% to 15%, is characterised mainly by excessive androgen levels, is accompanied by obesity and insulin resistance (IR), and affects female ovulation function and the conception rate [1][2][3]. At present, its pathogenesis is still unclear. High levels of androgens, the environment, and family genetics may all be related to PCOS [4]. Recent studies [5][6][7] have shown that a deficiency in 25-hydroxyvitamin D[25(OH)D] is associated with sex hormone secretion and glucose metabolism disorders in patients with PCOS. The transforming factor superfamily includes Müllerian Inhibiting Substance (MIS). An overabundance of MIS may impact dominant follicle development. Insulin-like growth factor 1 (IGF-1) is a vital regulatory component that helps regulate protein, lipid, and carbohydrate metabolism. It also has a connection to follicular development and the production of sex hormones [8][9][10]. Insulin-like growth factor binding protein 3 is the main protein that regulates islet growth factor (IGFBP-3). It can bind to IGF-1, prolonging its half-life, and is associated with the suppression of follicular development [11].

A prevalent endocrine condition in women with a comparatively high incidence rate, polycystic ovarian syndrome (PCOS) is intimately linked to several health issues, such as infertility, menstrual disorders, and metabolic abnormalities [12]. In recent years, with increasing understanding of PCOS, research has revealed that its aetiology is complex and involves multiple factors, such as genetics, the environment and hormone levels. A variety of serum biomarkers, including 25(OH)D, MIS, IGF-1, and IGFBP-3, have been proposed as potential diagnostic indicators of PCOS [13]. However, there is still some controversy over their application value in the diagnosis of PCOS. 25(OH)D, the main metabolite of vitamin D, is closely associated with metabolic abnormalities and hormonal imbalances in PCOS [14]. MIS, a marker of ovarian function, usually increases in patients with PCOS. IGF-1 and IGFBP-3 regulate growth factors and hormones in the body and may play important roles in the development and progression of PCOS [15]. Therefore, exploring the diagnostic value of these biomarkers in PCOS is clinically significant. It not only helps in early disease diagnosis but also provides a theoretical basis for the formulation of individualised treatment plans.

Our goal was to provide more precise diagnostic techniques for clinical practice by further evaluating the diagnostic potential of serum levels of 25(OH)D, MIS, IGF-1, and IGFBP-3 in PCOS.

### Materials and methods

### General information

212 PCOS patients who presented to our institution between May 2022 and May 2024 were selected as the observation group.

Inclusion criteria: (1) Met the diagnostic criteria for PCOS and (2) Did not receive treatment within 3 months before enrollment.

Exclusion criteria: (1) Severe organic damage to the liver or kidneys exists; (2) Endocrine diseases not caused by PCOS; (3) Recently taken drugs that affect the levels of 25(OH)D, MIS, IGF-1 and IGFBP-3; (4) some tumours secrete androgens; (5) Combined acute and chronic infections. An additional 212 healthy women who visited our hospital for physicals at that time were selected as the control group.

### Serum levels of 25(OH)D, MIS, IGF-1 and IGFBP-3

Early in the morning of the third to fifth day of the menstrual cycle, 3 mL of fasting venous blood was drawn from each of the two subject groups. The enzyme-linked immunosorbent test was used to evaluate the levels of IGF-1, IGFBP-3, 25(OH)D, and blood MIS (ELISA).

### Sex hormones and glucose metabolism indicators

An automatic electrochemiluminescence immunoassay analyser was used to measure the amounts of estradiol (E2), total testosterone (TT), follicle-stimulating hormone (FSH), luteinising hormone (LH), fasting insulin (FINS), and fasting blood glucose (FBG) in the serum of the two subject groups. The formula for calculating the homeostasis model insulin resistance index (HOMA-IR) is HOMA-IR=FINSxFBG/22.6. With a HOMA-IR score 3.8 as the diagnostic criterion for IR, the observation group was divided into two subgroups: the IR group (148 patients) and the non-IR group (64 patients).

### Laboratory testing methods

All fasting venous blood samples collected from the research subjects in the early morning in this study were left to stand at room temperature, then centrifuged (3000 rpm for 10 minutes) to separate the serum. The target serum markers were determined in strict accordance with the instructions of each test item using a routine clinical detection platform.

A chemiluminescence immunoassay (CLIA) was used to measure the levels of anti-Mullerian hormone (MIS) and blood 25-hydroxyvitamin D [25(OH)D], among which the detection of 25(OH)D was carried out via the Roche Cobas e601/e602 electrochemiluminescence immunoassay system and its matching reagents from Roche Diagnostics, item no. 07092707 190; MIS detection was carried out via the Beckman Coulter Access 2/Access DxI 800 system and the matching reagents (Manufacturer: Beckman Coulter, item number: B13148). The concentrations of insulin-like growth factor-1 (IGF-1) and its binding protein-3 (IGFBP-3) were determined via enzyme-linked immunosorbent assay (ELISA). IGF-1 detection was performed with a DSL kit (Manufacturer: DSL/now under Beckman Coulter, item number: 10-5600), and IGFBP-3 detection was performed with a DSL kit (manufacturer: DSL/Beckman Coulter, item number: 10-6600). All testing is carried out under strict indoor quality control conditions and performed by professional inspectors. The interpretation and recording of results are subject to a double-person verification system to ensure the accuracy and reliability of the testing data.

### Statistical processing methods

SPSS 20.0 was used to analyse the data. Measurement data that fit a normal distribution are reported as x̄±s, and the t-test was used for all group comparisons. The associations between serum 25(OH)D, MIS, IGF-1, and IGFBP-3 levels and HOMA-IR were examined using Pearson correlation analysis. Serum 25(OH)D, MIS, IGF-1, and IGFBP-3 were evaluated for their auxiliary diagnostic efficacy in the treatment of PCOS using receiver operating characteristic (ROC) curves. A P value below 0.05 was regarded as an indicator of statistical significance.

## Results

### Comparison of general characteristics between the two groups

There was no statistically significant difference in the average age between the observation group (27.16±3.07 years) and the control group (26.47±3.29 years) (P>0.05). However, the average BMI in the observation group (27.14±3.44 kg/m^2^) was significantly greater than that in the control group (23.64±4.15 kg/m^2^) (P<0.05).

### The levels of serum 25(OH)D, MIS, IGF-1 and IGFBP-3 in the two groups

Although the observation group's serum 25(OH)D level was lower than the control group's, their levels of serum MIS, IGF-1, and IGFBP-3 were higher. The differences were statistically significant, as demonstrated by P<0.05.

According to the subgroup analysis of PCOS patients, the above differential characteristics of insulin resistance (IR) patients were more significant: the 25(OH)D level in the IR subgroup was considerably lower than that in the non-IR subgroup, although the levels of MIS, IGF-1 and IGFBP-3 were significantly greater than those in the non-IR subgroup. Correlation analysis further revealed that MIS, IGF-1 and IGFBP-3 were positively correlated with the insulin resistance index (HOMA-IR), whereas 25(OH)D was negatively correlated with HOMA-IR (Table 1).

**Table 1:** Comparison of serum levels of 25 (OH) D, MIS, IGF-1, and IGFBP-3 between two groups (x̄±s).

Group	n	25(OH)D (g/L)	MIS (ng/mL)	IGF1 (g/L)	IGFBP-3 (μg/L)
Observation group	212	28.96±5.37	8.15±1.69	271.25±52.23	63.59±8.48
Control group	212	40.72±6.58	3.55±104	202.35±49.67	51.25±10.59
t		9.996	16.763	6.899	6.585
P		<0.001	<0.001	0.009	<0.001

### Sex hormones and markers of glucose metabolism between the two groups

The observation group's levels of LH, E2, TT, FBG, FINS, and HOMA-IR were all considerably higher than the control group's (P<0.05). The two groups' FSH levels did not differ statistically significantly (P>0.05).

Patients with polycystic ovary syndrome (PCOS) exhibit characteristic changes in sex hormone profiles and glucose metabolism homeostasis. The levels of serum luteinising hormone (LH), estradiol (E2), and total testosterone (TT) in the observation group were significantly increased, indicating the typical pathological features of hypothalamic pituitary ovarian axis dysfunction and hyperandrogenemia. In terms of glucose metabolism, fasting blood glucose (FBG), fasting insulin (FINS), and the insulin resistance index (HOMA-IR) are elevated simultaneously in patients with PCOS, suggesting that their abnormal glucose tolerance and insulin resistance are significantly more severe than those of healthy individuals. Sex hormone abnormalities and glucose metabolism disorders showed a synchronous strengthening trend in the observation group, reflecting the multiple endocrine and metabolic imbalance characteristics of PCOS patients (Table 2).

**Table 2:** Comparison of sex hormones and glucose metabolism indicators between the two groups (x±s).

Group	n	FSH <br>(mU/mL)	LH <br>(mU/mL)	E_2_ <br>(pmol/L)	TT <br>(nmol/L)	FBG <br>(mmol/L)	FINS <br>(mU/L)	HOMA-IR
Observation group	212	5.98±1.41	8.44±2.05	157.39±34.22	3.06±0.77	5.25±0.46	15.44±3.15	4.18±0.99
Control group	212	6.25±1.53	3.56±1.15	134.18±31.25	1.34±0.39	4.52±0.47	6.06±1.28	2.04±0.45
t		0.94	14.908	3.616	14.685	8.047	20.118	13.854
P		0.37	<0.001	0.001	<0.001	<0.001	<0.001	<0.001

### Comparison of serum 25(OH)D, MIS, IGF-1 and IGFBP-3 levels between the IR group and the non-IR group

While the serum levels of MIS, IGF-1, and IGFBP-3 were higher in the IR group than in the non-IR group, the serum 25(OH)D level was lower in the IR group. P<0.05 indicated that the differences were statistically significant.

The 25(OH)D level in the insulin-resistant group was significantly lower than that in the non-insulin-resistant group, while the levels of MIS, IGF-1 and IGFBP-3 were greater. These variations imply that the levels of these markers in PCOS patients may be significantly impacted by insulin resistance. Additionally, there was a positive association between the insulin resistance index and MIS, IGF-1, and IGFBP-3, but a negative correlation with 25(OH)D. These findings suggest that insulin resistance contributes significantly to the onset and progression of PCOS, and identifying these indicators helps better understand its clinical characteristics (Table 3).

**Table 3:** Comparison of serum levels of 25 (OH) D, MIS, IGF-1, and IGFBP-3 between the IR group and non-IR group (x±s).

Group	n	25(OHD (pg/L)	MIS (ng/mL)	IGF-1 (ng/mL)	IGFBP-3 (ng/mL)
IR group	148	27.16±5.28	9.07±1.53	281.29±51.32	65.18±9.59
Non IR group	64	33.19±5.19	6.06±0.97	248.07±50.12	59.88±10.23
t		5.479	10.424	3.077	2.578
P		<0.001	<0.001	0.003	0.011

### Correlation analysis of serum 25(OH)D, MIS, IGF-1 and IGFBP-3 levels with HOMA-IR in the observation group

Serum 25(OH)D had a negative correlation with HOMA-IR (P<0.05), while serum levels of MIS, IGF-1, and IGFBP-3 were favorably linked with HOMA-IR (P<0.05) in the observation group.

The insulin resistance index (HOMA-IR) showed a strong correlation with the observation group's serum 25(OH)D, MIS, IGF-1, and IGFBP-3 levels. There is a negative correlation between HOMA-IR and serum 25(OH)D levels. Serum 25(OH)D levels were lower in those with more severe insulin resistance. MIS, IGF-1, and IGFBP-3 levels were strongly correlated with HOMA-IR, indicating that patients with higher hormone levels had more severe insulin resistance. These findings imply that the insulin resistance mechanism of PCOS may involve significant roles for serum 25(OH)D, MIS, IGF-1, and IGFBP-3 (Table 4).

**Table 4:** Correlation analysis between serum 25 (OH) D, MIS, IGF-1, IGFBP-3 levels and HOMA-IR in the observation group.

Indicator	HOMA-IR	
	r	P
25(OH)D	-0.545	<0.001
MIS	0.659	<0.001
IGF-1	0.582	<0.001
IGFBP-3	0.567	<0.001

### Analysis of the auxiliary diagnostic value of serum 25(OH)D, MIS, IGF-1 and IGFBP-3 for PCOS

The results of the ROC curve analysis revealed that the area under the curve (AUC) for the combined auxiliary diagnosis of PCOS based on serum 25(OH)D, MIS, IGF-1, and IGFBP-3 was 0.963, which was greater than that for the individual detection of each index (Table 5).

**Table 5:** Analysis of the auxiliary diagnostic value of serum 25 (OH) D, MIS, IGF-1, and IGFBP-3 in PCOS.

Indicator	AUC	P	Truncated value	Sensitivity (%)	Specificity (%)	95% CI
25(OH)D	0.808	<0.001	33.88 μg/L	78.32	76.44	0.752~0.867
MIS	0.864	<0.001	5.49 ng/mL	86.77	90.59	0.804~0.919
IGF-1	0.813	<0.001	239.35 μg/L	76.46	74.57	0.755~0.875
IGFBP-3	0.799	<0.001	57.59 μg/L	74.55	75.45	0.736~0.858
25(OH)D+MIS + <br>IGF-1+IGFBP-3	0.963	<0.001	-	87.75	96.28	0.935~0.989

These four markers are strongly associated with the formation and progression of PCOS, according to our study of the participants' test results. In particular, MIS, IGF-1, and IGFBP-3 are closely associated with insulin resistance and are positively correlated with HOMA-IR, whereas 25(OH)D is negatively correlated with HOMA-IR. Further analysis of the subjects' operating characteristics indicated that the diagnostic value of combined detection of these four biochemical markers was much greater than that of individual detection of each marker. The combined detection's area under the curve was significantly larger than each indicator's, according to an analysis of the receiver operating characteristic (ROC) curve, indicating that this combined detection scheme can provide a more sensitive and specific reference for the clinical diagnosis of PCOS.

## Discussion

PCOS is an endocrine and metabolic disorder that can cause polycystic changes in the ovaries, menstrual disorders, hyperandrogenism and other symptoms and can affect normal conception [16]. Studies [17][18][19] have shown that 50% to 70% of PCOS patients have IR, with elevated androgen levels that affect follicular development. 25(OH)D is an intermediate product of vitamin D metabolism. It is linked to coronary heart disease, diabetes, and other illnesses, and it can regulate the differentiation of human reproductive cells and maintain calcium and phosphorus homeostasis [20]. Relevant studies [21][22][23] suggest that 25(OH)D has a certain relationship with PCOS. When the concentration is 20 ng/mL, the ovulation rate significantly increases. The observation group in this study had lower 25(OH)D levels than the control group, and the IR group had lower 25(OH)D levels than the non-IR group [24]. Additionally, there was a negative correlation between blood 25(Oh)D levels and the HOMA-IR score, indicating that PCOS patients had lower 25(OH)D levels than healthy individuals and that these levels were associated with the development of IR. Studies have shown that 25(OH)D can stimulate estrogen and progesterone production, affect insulin -cell function, and thereby influence the progression of IR [25]. Other studies have shown that supplementing with 25(OH)D in patients with PCOS can significantly improve insulin sensitivity [26].

MIS can be used to assess ovarian reserve function and has an inhibitory effect on follicular development [27][28]. Moreover, the serum MIS level was positively correlated with HOMA-IR. The interaction between serine threonine kinase type I and type 2 receptors in the ovary activates MIS activity [29]. MIS can inhibit steroid synthesis, activate the hypothalamus to release GnRH, inhibit the conversion of androgens to estrogens, cause follicular development disorders, and trigger PCOS [30].

The liver cells produce and release the peptide hormone known as IGF-1. It is crucial to the body's growth, development, and metabolism and shares structural and functional similarities with insulin. In the serum and ovaries, IGFBP-3 is the most prevalent IGF-binding protein [31]. It can form a complex with IGF-1, thereby affecting its biological function [32]. Because insulin levels in PCOS patients can stimulate increases in IGF-1 and IGFBP-3, and because signal transmission between insulin and IGF-1 receptors is impaired, this can lead to IR in PCOS patients. IGFBP-3 binds the same site as the -transcription factor retinoic acid X receptor (RXR) and can alter glucose homeostasis via RXR, thereby contributing to IR development in PCOS. Relevant studies [33][34] have shown that IGFBP-3 levels can serve as a diagnostic marker and a reference for clinical diagnosis and treatment, as they are closely linked to glycolipid metabolism in obese PCOS patients. This value was higher than the individual detection of each index. This study also has certain shortcomings. Given the small sample size, there may be some bias. In the future, we will collaborate with multiple centres to expand the sample size and enhance the richness and accuracy of this study.

## Conclusion

MIS, IGF-1, IGFBP-3, and serum 25(OH)D are associated with the development and occurrence of PCOS. Combined detection has high auxiliary diagnostic value for PCOS and is conducive to its clinical prevention and treatment.

## Dodatak

### Funding

None.

### Acknowledgments

None.

### Conflict of interest statement

All the authors declare that they have no conflict of interest in this work.
